# Synthesis and Applications of Graphene Oxide

**DOI:** 10.3390/ma15030920

**Published:** 2022-01-25

**Authors:** Adéla Jiříčková, Ondřej Jankovský, Zdeněk Sofer, David Sedmidubský

**Affiliations:** Department of Inorganic Chemistry, Faculty of Chemical Technology, University of Chemistry and Technology, Technická 5, 166 28 Prague 6, Czech Republic; Adela.Jirickova@vscht.cz (A.J.); Ondrej.Jankovsky@vscht.cz (O.J.); Zdenek.Sofer@vscht.cz (Z.S.)

**Keywords:** graphene oxide, synthesis, characterization, applications

## Abstract

Thanks to the unique properties of graphite oxides and graphene oxide (GO), this material has become one of the most promising materials that are widely studied. Graphene oxide is not only a precursor for the synthesis of thermally or chemically reduced graphene: researchers revealed a huge amount of unique optical, electronic, and chemical properties of graphene oxide for many different applications. In this review, we focus on the structure and characterization of GO, graphene derivatives prepared from GO and GO applications. We describe GO utilization in environmental applications, medical and biological applications, freestanding membranes, and various composite systems.

## 1. Introduction

Graphene is one of the most studied materials in the world; thanks to its unique properties, it was called a “material of the future” [1]. Graphene consists only of carbon atoms where every carbon atom is attached to three other carbon atoms with sp^2^ hybridized orbitals making a honeycomb lattice [2]. Graphene’s rare properties make it a very promising material for a huge variety of applications, including field-effect transistors (FETs), gas and biomolecules sensors, transparent conductive films (TCFs), and graphene batteries [3,4,5,6,7].

Graphene oxide (GO) is a layered carbon structure with oxygen-containing functional groups (=O, -OH, -O-, -COOH) attached to both sides of the layer as well as the edges of the plane [8]. As with any 2D carbon material, GO can also have either single layer or multilayer structure. A structure with one layer is graphene oxide; two layers of graphene oxide are referred to as a two-layered GO. GO with more than two layers and less than five layers is called few-layered graphene oxide, GO with five to ten layers is called multilayered GO, and material with eleven or more layers is called graphite oxide [9]. GO can be synthesized by the oxidation of graphite into graphite oxide followed by the exfoliation of this graphite oxide into GO. The properties of the material are strongly dependent on the synthesizing method, which influences the resulting number and type of oxygen-containing groups in the formed GO. In contrary to graphene, GO is hydrophilic, and it is hence relatively simple to prepare a water- or organic solvent-based suspensions. Highly oxidized forms of GO are electric insulators with a bandgap of approximately 2.2 eV.

**Figure 1:**
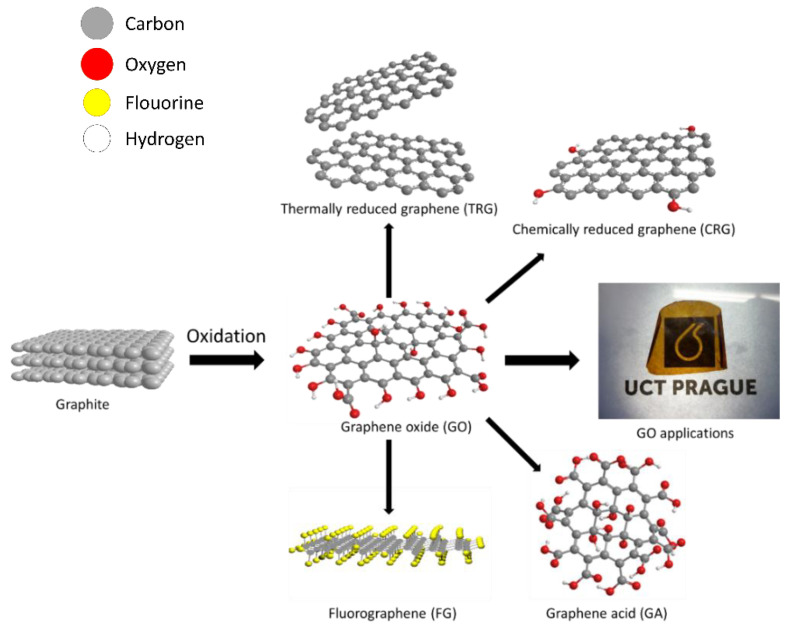
Scheme of preparation and utilization of GO (colors of atoms: grey—carbon, red—oxygen, yellow—fluorine, white—hydrogen).

Due to the presence of various oxygen functionalities on the surface of GO, GO can be used as a starting material for the synthesis of graphene derivatives such as fluorographene, bromographene, graphane, and many others. On the other hand, by thermal or chemical reduction of GO, thermally or chemically reduced graphene can be prepared (see Figure 1). Interestingly, GO can also be used for advanced applications such as for drug delivery, in high-temperature materials, or in construction materials. There are still some remaining issues that can be improved and studied more intensively. It is very important to develop novel methods of environmentally friendly low-cost large-scale synthesis of GO. In this review, we tried to summarize available knowledge about GO structure, synthesis and characterization of GO, GO functionalization, and selected GO applications.

## 2. Structure of GO

Over the years, the structure of GO was studied in detail using several instrumental techniques: annular dark-field imaging, ^13^C and ^1^H NMR, ultra-high-resolution transmission electron microscopy, X-ray diffraction, and many others [10,11,12,13]. Despite the number of attempts to reveal the structure of GO, a number of possible structural models exist with no unambiguous one. The main reason for this is the complexity of the material and the originality of every sample with variable stoichiometry [14]. Simplistically, GO is a monolayer sheet of graphite containing hydroxyl, carboxyl, and epoxy oxygen groups on its basal plane and edges, resulting in a mixture of sp^2^ and sp^3^ hybridized carbon atoms [15].

**Figure 2:**
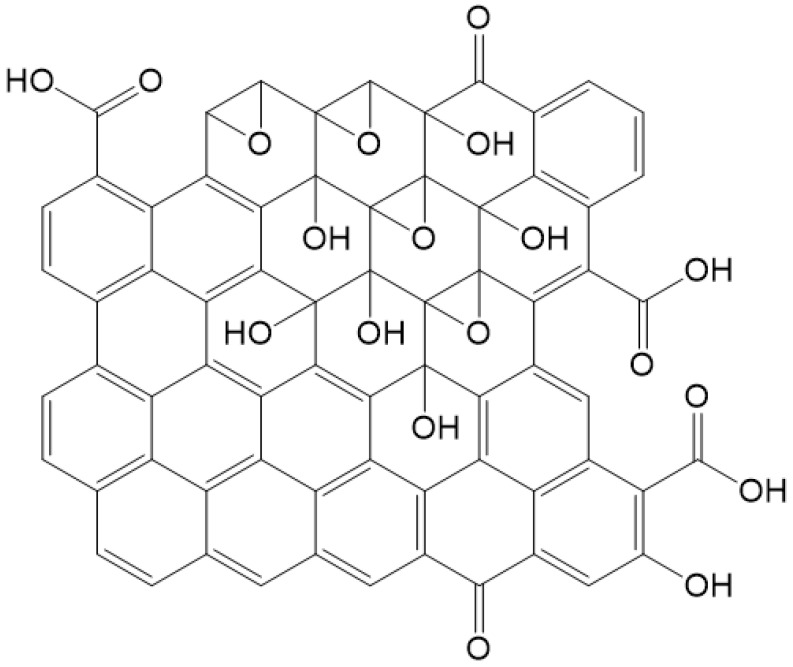
Lerf–Klinowski model of graphene oxide.

Many models of GO have been developed based on a number of analyses and theoretical simulations. The first model was suggested by Hofmann and Rudolf [16] in 1939, where a lot of epoxy groups were distributed randomly across the graphite monolayer. Then, in 1946, Ruess [17] updated the model by incorporating hydroxyl groups and alternating sp^2^ hybridized carbons with those revealing sp^3^ hybridization. In 1969, Scholz and Boem [18] suggested a less organized structure with double bonds C=C and periodically recurring C-C single bonds in the carbon layers that are corrugated with hydroxyls and carbonyls, without ether oxygen. Later, in 1994, Nakajima and Matsuo [19] proposed a model that resembled graphite intercalation compound. Then, in 1998, Lerf and Klinowski [11] created a model (LK model) that contains two different kinds of regions: regions with six-membered aliphatic rings and regions with nonoxidized benzene aromatic rings (see Figure 2). The size of the two regions is dependent on the level of material oxidation. The model is composed mainly of aromatic bodies, epoxide groups, and double bonds. Wrinkling in the monolayer is caused by the slightly distorted tetrahedral configuration of hydroxyl groups attached to carbon atoms. The oxygen functional groups are attached to the monolayer of carbon above and below, creating two layers of oxygen atoms with variable concentrations composed mainly of epoxide and hydroxyl groups that are very close to each other. All of the oxygen functionalities, aromatic bodies, and oxidized rings are distributed randomly across the carbon monolayer. The acidity of GO can be explained by the oxygen groups that are attached to the edges of the lattice, which are hydroxyl and carboxyl groups. This LK model has become one of the most acceptable and used models for moderately oxidized GO.

After the discovery of graphene, researchers worldwide started to focus on GO and other derivatives. In 2006, Szábó and Dékány examined previous models by a number of analyses and suggested a model without carboxylic acids composed of two main regions: corrugated hexane ribbons occupied with quinones and ketones, and translinked cyclohexane chairs with 1,3-epoxide and tertiary alcohols. In 2013, Dimiev, Alemany, and Tour [20] proposed a dynamical structural model (DSM) that describes the development of various carbon structures with attached water, contrary to the static LK model. Recently, Liu et al. [21] experimentally observed the evidence of the C=O bonds on the edge of the carbon monolayer, confirming parts of the earlier models, especially the LK model [22]. Real GO also includes some defects, such as topological defects (pentagons, heptagons, octagons, etc.), adatoms, vacancies, and adsorbed impurities.

By using concentrated acids for oxidation, low-molecular-weight fragments are produced, known as oxidation debris [23,24]. Oxidation debris is a mixture of highly oxidized polyaromatic fragments adsorbed on the poorly oxidized GO platelets by π–π stacking, hydrogen bonding, and van der Walls interactions [25]. The amount of the oxidation debris is strongly influenced by the reaction time of graphite and concentrated acids [26,27]. To wash away those fragments, a base washing is needed [27,28,29]. The pure GO without the oxidation debris presents an oxidation level that is similar to chemically reduced GO [30].

## 3. Conventional Routes of GO Synthesis

The first attempt to synthesize graphite oxide was performed in 1859 by British chemist B. C. Brodie who investigated the reactivity of flake graphite [31,32]. It is a chlorate route, where potassium chlorate is used as an oxidizing agent. Benjamin Brodie treated graphite with a number of strong oxidizing agents for the first time to decode its structure. In the experiment, he treated graphite in a mixture of potassium chlorate and fuming nitric acid at 60 °C for 4 days (Brodie’s graphene oxide (BR-GO)). He performed multiple oxidative treatments one after another (4–7) and the resulting composition of carbon, oxygen, and hydrogen was estimated as C_11_H_4_O_5_ (corresponds to C/O ratio 2.2) [32]. The product was found to be soluble in pure water, while it tended to flocculate in a more acidic environment. Brodie named the product “graphic acid” because it had a slight reaction with litmus paper. Another chlorate route is the Staudenmaier method [33]. Later, L. Staudenmaier modified Brodie’s method by adjusting the way the chlorate was added and also adding sulfuric acid into the mixture (ST-GO—Staudenmaier’s graphene oxide). Potassium chlorate was added in small portions into the mixture in order to eliminate the danger of explosive by-products and heat evolution. The increased acidic environment caused a decrease in terms of reaction time. The obtained material has very similar properties to BR-GO. In 1937, Hofmann used potassium chlorate and nonfuming nitric acid to synthesize Hofmann’s graphene oxide (HO-GO) with lower oxygen content (C/O ratio 2.5). It was found that the concentration of nitric acid highly influences the level of oxidation of the resulting graphite oxide or graphene oxide [34]. The lower the concentration of nitric acid, the higher level of oxidation of graphene oxide.

The most used and effective method of all time is one of the permanganate methods, the Hummers method [35] created by Hummers and Offeman. It is a relatively fast conventional method used for the synthesis of GO. In this method, the reaction mixture is composed of an excess of potassium permanganate, sulfuric acid, and a small amount of sodium nitrate. The reaction time ranges between 8 and 12 h. This route is much safer because it avoids the creation of explosive ClO_2_. At the end of the reaction, the excess of the potassium permanganate is neutralized with a diluted solution of H_2_O_2_. The product of the Hummers’ method (Hummers’ graphene oxide (HU-GO)) has a very similar C/O ratio (2.25) to the C/O ratio of BR-GO (2.2). Unfortunately, this method is not environment-friendly, because of NO_x_ that evolves during the reaction. There are several modified Hummers methods, including nitrate-free [36,37,38], two-step [36,37], co-oxidant [38], and low- and room-temperature [39,40] methods. Then, in 2010, Tour developed his own method, Tour’s method, which is described below.

## 4. Modern Ways of GO Synthesis

There are several ways to prepare graphite oxide/graphene oxide. The most common way is to use an oxidizing agent in an acidic environment. Other methods are electrochemical and microbial.

**Figure 3:**
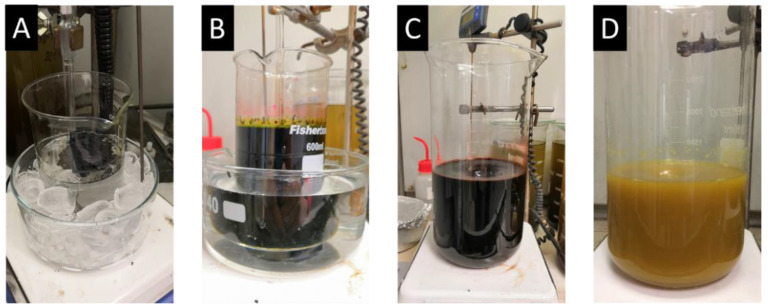
Photographs describing preparation process of GO by Tour’s method: (**A**) before addition of potassium permanganate; (**B**) after oxidation; (**C**) after pouring on ice; (**D**) after addition of H_2_O_2._

In 2010, a novel method was developed. Tour’s method [41] (Tour’s graphene oxide (TO-GO)) also falls under permanganate methods. In this procedure, phosphoric acid is mixed with sulfuric acid in the ratio 1:9 and potassium permanganate and graphite added in the ratio 6:1 in an ice bath (Figure 3A). The mixture is then heated at 50 °C and stirred for 12 h (Figure 3B). After cooling down, the mixture is poured onto ice (Figure 3C). Finally, 30% H_2_O_2_ is added in order to remove the excess of potassium permanganate (Figure 3D). Phosphoric acid works as a dispersive and etching agent, as well as a stabilizer of the oxidation process, which makes the synthesis of GO safe. This route produces a higher yield of GO with a higher level of oxidation and a more regular structure.

Besides permanganate and chlorate methods, there are more modern ways to oxidize graphite in order to prepare GO, including the use of potassium chromate in combination with perchloric or nitric acid [42] or under the Jones conditions [43,44]. Alternatively, less toxic potassium ferrate in sulfuric acid [45] can be applied for GO preparation. Contrary to these results, another study evidences why it is not possible to prepare GO by using potassium ferrate [46]. Moreover, graphite can be oxidized in water with H_2_O_2_ at 50 °C by Fe(VI) [47] or at 110 °C with benzoyl peroxide [48]. Let us note that GO prepared by chemical routes often displays a highly damaged structure due to the harsh acidic conditions of the synthesis as well as the presence of impurities. Such characteristics are indeed far from being optimal for electronics applications. Even though chemical, especially chlorate and permanganate, ways of preparation provide GO with poor electrical properties, the exploration is not at its end yet. For example, in 2017, Jankovský et al. modified Tour’s method and suggested that the shortened reaction time (from 12 h to 1 h) has no significant impact on the resulting material [49]. In 2018, Ranjan et al. [50] also modified Tour’s method and proposed the oxidation process of graphite flakes in permanganate (ratio 1:6) in a mixture of sulfuric and phosphoric acids (ratio 9:1) heated at 65 °C for 12 h. All of the chemicals were precooled at 5 °C.

Apart from the chemical routes, electrochemical synthesis represents another approach to GO synthesis that might be the key to large-scale production. Electrochemical production is more eco-friendly than chemical production due to reusing the electrolyte multiple times and minimal washing of the utensils [51,52]. The better quality of electrochemical GO (EGO), in contrast to standard procedures, can be explained by the use of aqueous electrolytes and no need for oxidizing agents, hence avoiding impurities [52]. Moreover, thanks to the variety of experimental setups, the level of oxidation and density of defects can be controlled.

Interestingly, the usage of biological systems to oxidize graphitic materials is very important to obtain eco-friendly graphene oxide. However, after the microbial cultivation, graphite is not homogeneously oxidized. Acidithiobacillus ferrooxidans or Pseudomonas have been tested [53] as oxidizing bacteria.

## 5. Derivatives of GO

Derivatives of graphene oxide are materials based on GO as a starting material. These involve graphene acid (GAF), a highly oxidized GO exhibiting a composition close to [C_1_(COOH)_1_]_n_; chemically reduced graphene oxide (CRG) and thermally reduced graphene oxide (TRG), which are reduced forms of GO with some remaining oxygen functionalities left in the structure; and fluorographene, a fluorinated form of graphene with composition [C_1_F_1_]_n_ (see Figure 1). The functionalization of graphene oxide is possible thanks to the presence of oxygen functionalities, unlike in other carbon nanomaterials.

**Figure 4:**
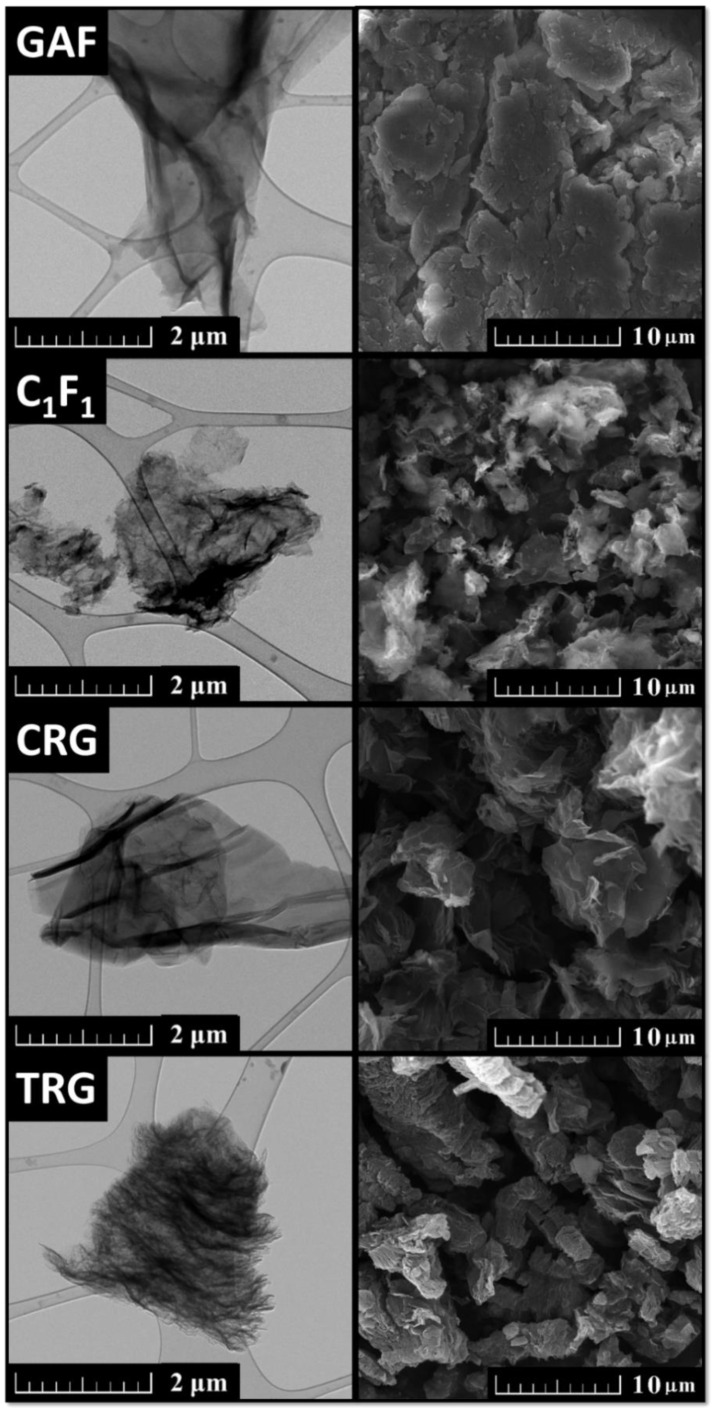
TEM (**left**) and SEM (**right**) micrographs of graphene acid (GAF), fluorographene (C_1_F_1_), chemically reduced graphene (CRG), and thermally reduced graphene (TRG).

**Figure 5:**
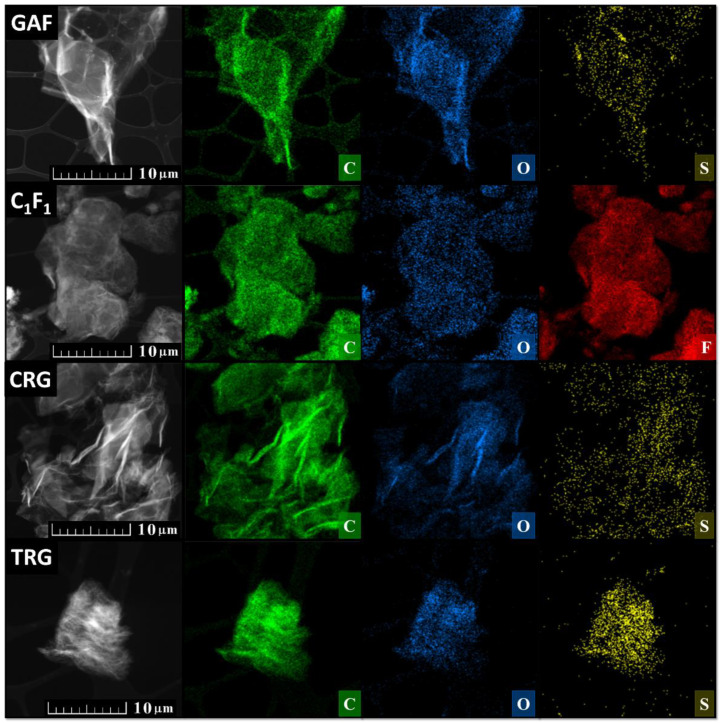
TEM and EDS micrographs of graphene acid (GAF), fluorographene (C_1_F_1_), chemically reduced graphene (CRG), and thermally reduced graphene (TRG).

For a closer inspection of the essential characteristics of those derivatives, we performed transmission electron microscopy (Figure 4 left), scanning electron microscopy (Figure 4 right), and energy-dispersive spectroscopy (Figure 5) to study the surface and composition of the samples. The first micrographs in Figure 4 of GAF show a wrinkled structure, where the flakes of GAF are connected together forming a foil-like structure. Next, micrographs of [C_1_F_1_]_n_ show small flakes of fluorographene which consist of multiple wrinkled sheets. Whereas micrographs of CRG do not show such wrinkled structure of multiple sheets, the last micrographs of TRG show small flakes of multiple-sheet structure which are highly wrinkled. According to EDS (see Figure 5), all samples consist of carbon, oxygen, and sulfur except for fluorographene which consists of carbon, oxygen, and fluorine.

Graphene acid (GA) is a graphene derivative with a composition close to [C_1_(COOH)_1_]_n_. The synthesis of such material consists of two consecutive oxidation steps of graphite. After the first oxidation by the Tour method, GO is obtained and further used as a starting material for the second oxidation. The second oxidation runs according to the Tour method as well [54]. Further oxidation leads to a total decomposition of GA (oxidation to CO_2_). Another possible way to synthesize GA is by acidic hydrolysis of cyanographene (graphene–nitrile) by 20% HNO_3_ [55].

GO is mostly used for the production of graphene (reduced graphene oxide (rGO)) by chemical (chemically reduced graphene (CRG)) or thermal (thermally reduced graphene (TRG)) reduction. Reduced graphene oxide can be used in electronic devices, energy storage devices, (bio)sensors, biomedical applications, supercapacitors, membranes, catalysts, and water purification. As an electronic device, rGO is used in field-effect transistors (FETs) as chemical sensors and biosensors [56,57,58,59]. rGO was also used in light-emitting diodes (LEDs) as a transparent electrode [60,61]. Thanks to the extreme surface area of rGO, the material is used as an electrode in double-layered capacitors, batteries, fuel cells, and solar cells [62,63]. Energy storage capacity and cycle stability of Li-ion battery devices can be enhanced using Fe_3_O_4_ on rGO anode rather than pure Fe_3_O_4_ or Fe_2_O_3_ [64]. Stacked sheets of GO have nanocapillaries between individual sheets, which are closed by chemical reduction of GO, creating a material that is impermeable to liquids, gases, and even strong chemicals. Corrosive acids can be stored in glass or copper containers that are covered inside with such graphene paint [65,66]. In order to improve shelf life in medical infrastructure, graphene-coated plastic films may be used [67].

Graphene oxide can be reduced thermally simply by applying heat, a process called thermal annealing reduction. Firstly, the exfoliation of GO occurs during rapid heating, where gases such as CO_2_, CO, and H_2_O are released from the sample [68,69,70]. During the rapid increase in temperature, the oxygen-containing groups transform into gases mentioned above that generate huge pressure between the stacked layers of graphene oxide. At approximately 300 °C, the pressure reaches ~40 MPa, and it is increased to ~130 MPa if the temperature is raised to 1000 °C [71]. In fact, a pressure as low as 2.5 MPa is enough to separate two stacked layers of GO, as predicted by the evaluation of the Hamaker constant [71]. The originated platelets can be referred to as graphene or TRG because the elevated temperature causes decomposition of oxygen-containing functional groups which leads to the exfoliation of the material. This could be a good strategy for bulk rGO synthesis; however, this route yields only small-sized and wrinkled graphene sheets [68,72]. This effect is caused by the removal of carbon atoms during the transformation of oxygen-containing groups into the above-mentioned gases, which splits wide sheets of graphene oxide into small-sized graphene sheets [73,74]. High temperatures of the thermal reduction lead to the emissions of highly toxic volatile organic hydrocarbons [75]. Another way to fabricate reduced graphene oxide is by a liquid-phase exfoliation in an inert atmosphere. This route is highly affected by the temperature of GO reduction [6,68,76,77]. At a temperature lower than 500 °C, the C/O ratio is not higher than 7, while when the temperature reaches 750 °C, the C/O ratio is very likely to be higher than 13. In addition to this effect, there is also a great importance of the used annealing atmosphere. Annealing reduction of GO can be carried out in vacuum [6], inert [77], or reducing atmosphere [69,77,78,79].

The first option to reduce GO chemically is using the reducing agent at room temperature or at slightly elevated temperature. It is an easy and cheap way for the mass production of graphene in comparison to the thermal reduction route. Hydrazine was the first chemical compound used for the reduction of GO even before the discovery of graphene [80]. Stankovich et al. reported the preparation of chemically derived graphene using hydrazine [81,82]. Apart from hydrazine, its derivatives such as hydrazine hydrate and dimethylhydrazine can be also used to reduce GO [83]. The reduction is achieved by adding the liquid reagents to a GO aqueous suspension, where the graphene-based nanosheets are agglomerated due to the increased hydrophobicity. Another great chemical reducing reagent is ascorbic acid (vitamin C), which is considered to be an ideal hydrazine substitute [84]. The resulting material has a very similar C/O ratio as the one reduced by hydrazine, but vitamin C has a great advantage of nontoxicity. Moreover, the colloid state reduction of vitamin C does not bring about a product agglomeration, which is helpful for further applications. In addition, using Ar^+^ ion irradiation of GO foils creates highly conductive graphene papers [85].

Fluorographene is fluorinated graphene with stoichiometry [C_1_F_1_]_n_ [86,87]. As with every graphene/graphene oxide derivative, it has extraordinary electronic, optical, physical, and chemical properties that make it one of the thinnest insulators with a wide electronic gap [88]. The preparation of such a material can be divided into two strategies. The first one is based on the exfoliation of bulk graphitic materials containing fluorine atoms, while the second strategy relies on the fluorination of graphene or graphene oxide with fluorinating agents. Exfoliation can be performed in a liquid phase or mechanically. In the liquid-phase exfoliation, a medium is used to weaken the van der Waals interactions between the layers, resulting in single- or few-layer fluorographene [89]. For the first time, sulfolane was used and the mixture of the solvent and bulk graphite fluoride was sonicated for 1 h at 50 °C [90]. Isopropanol [91], ethanol [92], acetonitrile [93], and chloroform [94] can be mentioned as other reported solvents for the exfoliation of bulk graphite fluoride. Another strategy to prepare fluorographene is to combine graphene with fluorination agents such as xenon difluoride in a reactor [95]. The process can be initiated by exposing the reactants to temperature, irradiation, or pressure. Fluorographite and fluorographene can be used as a precursor for the synthesis of highly hydrogenated graphene (graphane) [96].

## 6. Typically Used Analytical Methods for GO

In order to provide typical analytical results of GO, a sample of GO was prepared by modified Tour’s method [49] and analyzed by SEM, EDS, and TEM. Usually, Raman spectroscopy, XPS, XRD, XRF, EA, EDS, AFM, and STA-MS are also used for the characterization.

**Figure 6:**
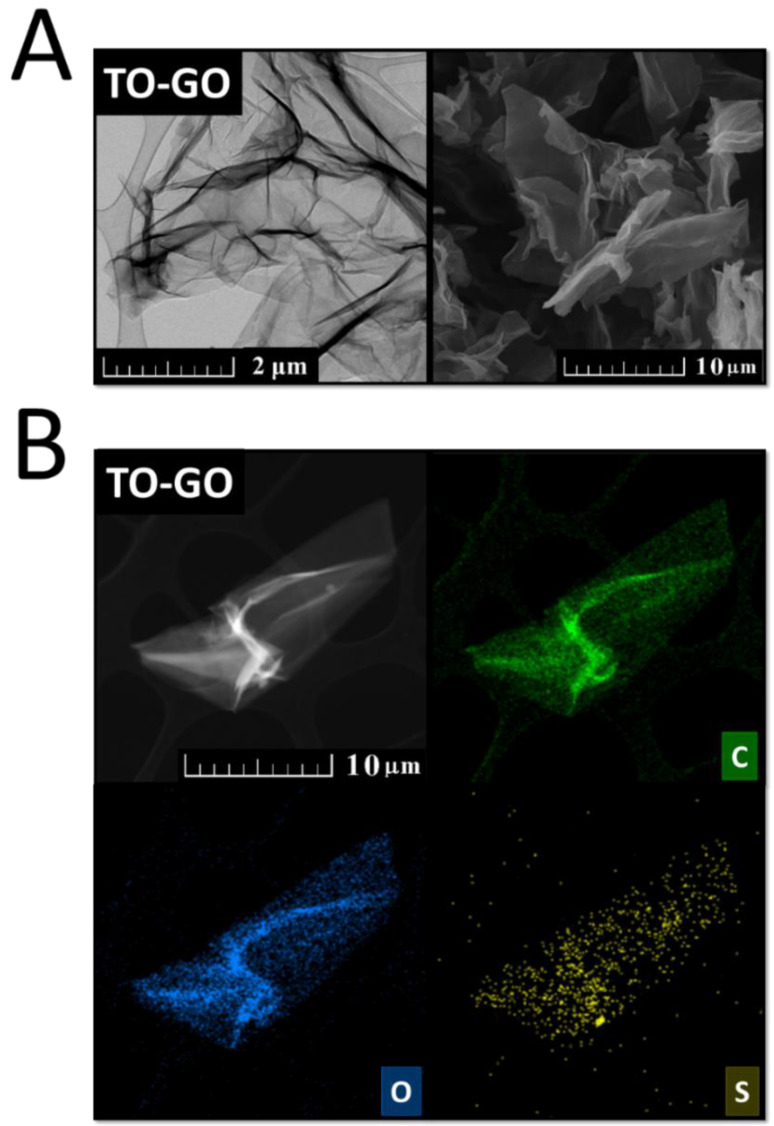
TEM (**left**) and SEM (**right**) micrographs (**A**) and EDS micrographs (**B**) of GO.

Transmission electron microscopy (TEM), energy-dispersive spectroscopy (EDS), and scanning electron microscopy (SEM) were used to study the surface of GO. The first micrographs in Figure 6A of TO-GO show wrinkled sheets of graphene oxide by both TEM and SEM methods. According to EDS (see Figure 6B), the sample consists of carbon, oxygen, and sulfur. Let us note that the hydrogen is not visible on EDS.

The composition of the sample can be determined via X-ray fluorescence (XRF). The composition of GO depends on the level of oxidation of the sample. Generally, graphene oxide is composed of carbon, oxygen, and hydrogen, but there are very often other impurities from the starting materials, such as sulfur, chlorine, nitrogen, manganese, and potassium [97]. Manganese and potassium are contaminants that remain from the starting reactants (potassium permanganate), and chlorine remains from hydrochloric acid that is usually used in order to wash away the contaminants.

The composition of TO-GO can be also investigated by elemental analysis (EA) [98]. The results can also prove the presence of impurities such as sulfur and nitrogen, depending on the used synthesis method. Sulfur and nitrogen remain from the sulfuric acid and nitric acid, respectively, that are used as starting chemicals to synthesize GO. EA is a suitable method to determine hydrogen content; however, the determination of oxygen is not precise due to the indirect calculation.

Raman spectroscopy is a powerful tool for the determination of defect rate. Using Raman spectroscopy, two local maxima around 1350 cm^−1^ and 1600 cm^−1^, respectively, are usually registered. The first local maximum is called the D band and shows a quasitetrahedral coordination with sp^3^ carbon hybridization (irregularities such as functional groups). The other local maximum is called the G band and detects a planar arrangement with sp^2^ hybridization (regular graphene lattice). The D/G ratio, calculated from the intensity of peaks, shows the level of oxidation of the sample and should be around 1.00 [99].

X-ray diffraction is used to determine the interlayer distance between GO layers. Whereas pure graphite has (002) reflection at 26.3° that corresponds to the interlayer distance of 3.342 Å [100], the (002) reflection for GO can be found around 10.0–12.0° [101,102,103,104,105], indicating an interlayer distance of around 7.4–9.0 Å. This significant increase in the interlayer distance of pristine graphite and graphene oxide is caused due to oxygen functional groups attached to the carbon layer.

GO may be analyzed by X-ray photoelectron spectroscopy (XPS) to determine the composition of the sample surface and more interestingly to determine the ratio of individual functional groups. Two local maxima are usually detected around ~284.4 eV for the C1s peak and around ~532.4 eV for the O1s peak. Maxima for N1s, S2s, and S2p peaks might also be found, showing the contamination of the sample by sulfur and nitrogen. The composition is very variable and depends on the level of oxidation of the sample [106]. The deconvolution of the C1s peak can be used in order to quantify individual bonds that are present in the samples, for a sample of deconvoluted C1s peak. From the C1s peak, six kinds of bonds can be distinguished: C–C at ~284.5 eV; C=C at ~285.2 eV; C-O at ~286.2 eV; C=O at ~287.8 eV, O–C=O at ~289.0 eV; and π–π* interaction at ~291.0 eV. From the O1s peak, four kinds of bonds with the following binding energy may be identified: O-C=O at ~531.2 eV, C=O at ~ 532.5 eV, C-OH at ~533.3 eV, and C-O-C at ~534.0 eV [107].

With simultaneous thermal analysis in an inert atmosphere, the temperature of exfoliation is investigated. The literature claims that the temperature of the exfoliation of graphene oxide is around 200 °C [108]. The obtained value is dependent on the heating rate and other conditions. The process of exfoliation, where stacked layers of graphene oxide are divided into individual reduced graphene oxide sheets, is accompanied by the formation of gases; gases detected by mass spectrometer were H_2_O, CO, and CO_2_.

**Figure 7:**
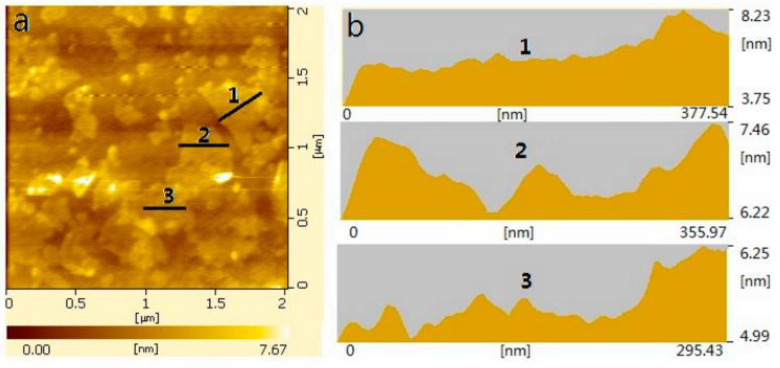
Typical AFM image of GO (**a**); surface patterns from AFM in positions 1, 2, and 3 (**b**) [111] (with the approval of MDPI *Materials*).

Atomic force microscopy (AFM) can be employed in order to study the thickness of the GO sample, therefore investigating the number of layers. It is known from the literature that a monolayered sample of GO has a thickness of 0.8–1.2 nm [109,110]. Shenghua Lv et al. reported AFM results of graphene oxide nanosheets with a sample thickness of less than 7.7 nm (see Figure 7).

## 7. Applications of GO

### 7.1. Environmental Applications of GO

One of the biggest threats to the environment is air pollution caused by the industrial release of harmful gases such as CO_2_, CO, NO_2_, and NH_3_. Thanks to the oxygen groups decorating the basal planes and the edges, GO is capable of covalent or noncovalent interactions with various molecules. GO can be employed in catalysis for converting polluting gases during industrial processing. The elimination of such harmful gases can be performed by capturing and storing gases, catalyst reactions of gas conversion, or direct utilization [112]. Apart from gas pollution, water pollution also represents a very huge environmental problem. The approach of GO application in this area can be divided into two paths: pollutant adsorption and conversion. The main water pollutants are heavy metal ions and organic dyes; they strongly threaten humans, aquatic life, animals, and plants.

#### 7.1.1. Removal of Toxic Gases 

The functional groups of few-layered GO composites exhibit unique adsorption behavior towards CO_2_ [113,114,115,116]. Laminar GO structures were assembled having fast and selective channels for gas separation with excellent preferential CO_2_ permeation performance [117]. Multi-permselective mixed matrix membranes and other mixed matrix membranes for efficient separation of CO_2_ were developed, enhancing the diffusivity selectivity, solubility selectivity, and reactive selectivity [118,119,120,121,122]. In addition, GO-based composites have a unique ammonia adsorption capability [123,124,125,126,127]. Moreover, other harmful gases such as acetone [128,129], formaldehyde [130], H_2_S [131,132,133], SO_2_ [134,135,136], and NO_x_ [137,138] can be adsorbed by GO-based composites.

#### 7.1.2. Conversion of CO_2_

Thanks to the unique electronic properties, GO-based composites exhibit superior photocatalyst abilities for CO_2_ conversion [139,140]. Hsu et al. [141] reported the photocatalytic conversion of carbon dioxide to hydrocarbons or their derivatives such as methanol for possible simultaneous CO_2_ reduction and solar energy harvesting.

#### 7.1.3. Water Purification

GO exhibits high adsorption ability towards Cd(II), Co(II), Au(III), Pd(II), Ga(III), and Pt(IV) [142,143,144]. Researchers Klímová et al. explored the adsorption ability of GO towards the whole periodic table. Adsorption ability mainly depends on the synthesizing method [145]. Few-layered graphene oxide nanosheets show a very high affinity towards Pb(II) ions, with a sorption capacity of about 842 mg g^−1^ at 293 K [146]. On the other hand, the adsorption capacity of Cu^2+^ ions is very low, even with oxygen groups on GO acting as active sites [147]. With the assistance of organic compounds, GO can provide more feasible anchoring sites for heavy metal ions [148,149,150,151,152,153,154,155]. Additionally, graphene oxide provides the ability to adsorb other harmful water pollutants—organic dyes [156,157,158,159]. Molla et al. [160] reported that the selectivity of positive dye methylene blue and rhodamine B was rapid (within 15 min) with the efficiencies of 97% and 88%, respectively, whereas the negative dye, methyl orange, was not absorbed.

### 7.2. Medical and Biological Applications of GO

The first possible application in this field is GO-based biosensors. GO-based biosensors rely on their preferred interaction with single-strand DNA (ssDNA) rather than double-strand DNA (dsDNA). This effect is caused by the effective hiding of nucleo-bases in dsDNA in a helical structure, which prevents GO from direct interaction with nucleo-bases [161,162,163].

Another interesting application is gene delivery, which is a promising way to treat genetic disorders, including cancer. The therapy uses gene vectors protecting DNA from nuclease degradation. GO sheets have been covered by polyethyleneimine (PEI) as a surface modifier for gene delivery into the cells. The delivery runs through complexation by electrostatic interaction and covalent conjugation for the loading of plasmid DNA (pDNA) [161,164].

Small-molecule drug delivery seems to be another promising medical application of GO. Small molecules of drugs can be attached to a GO surface using pH-sensitive linkers. A complex of doxorubicin and GO (DOX-GO) shows a release of DOX from GO dependent on pH due to higher solubility of DOX at low pH [165]. Moreover, cancer-targeting was successfully manifested as a codelivery of camptothecin (CPT) using folic acid conjugated nano-GO (FA-NGO) [166].

### 7.3. GO Membranes

**Figure 8:**
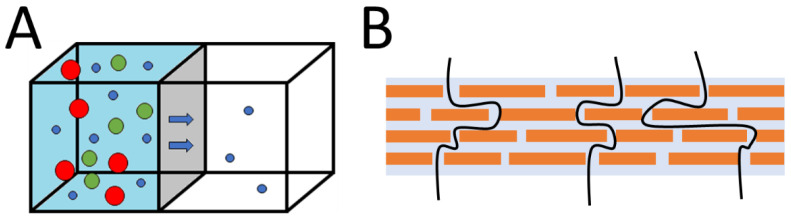
Membrane system scheme (**A**); schematic of possible water transport mechanism (**B**).

GO membranes may be used as ionic and molecular sieves or for selective gas transport. GO membranes were first introduced to the world by Nair et al. [167]. It was reported that a membrane of pure graphene oxide can block everything except for water vapor (see Figure 8A). Nair et al. claimed that a GO membrane allows only water vapor to pass through (Figure 8B), while ethanol and other alcohol molecules are blocked from passing through. The membrane can be prepared by vacuum filtration or by spraying a suspension of GO on a solid surface and then etching away the membrane from the surface. Others reported a study exploring the dependence of gases that pass through on the number of layers of GO. In other words, the selective diffusion of gases can be accomplished by the regulation of gas flow pores and channels by various stacking strategies [113].

As a reaction to Nair’s paper, Sun et al. [168] demonstrated a selective ion penetration through the GO membrane. They reported that salts of heavy metals and organic pollutants take a much longer time to permeate than sodium salts that pass through quite freely. The reason for this observation is that the salts of metals pass through capillaries in the membrane, where the heavy metal ions create coordination between the GO membrane and those ions that block the permeation. In 2014, Gao et al. [169] proposed the use of an ozonated GO membrane, having more oxygen functional groups, to improve proton conductivity in fuel cell applications at higher humidity. The water surface was used as a template for assembling the GO film on it by using the amphiphilicity of the GO [170].

### 7.4. High-Temperature Materials and GO

Graphene and graphene oxide are very promising materials for the reinforcement and general enhancement of mechanical properties of high-temperature materials. Some researchers studied the effects of graphene oxide on high-temperature materials such as metal alloys and ceramics. The mechanical resistance can be significantly improved by only 1 vol.% of GO. High energy ball milling was used to disperse graphene oxide powder in an aluminum (AlMg5) alloy matrix. Hot pressing was used to densify the obtained material [171]. More frequently, the reinforcement of high-temperature ceramics or graphene oxide/reduced graphene oxide coatings has been found to achieve better corrosion resistance. Spark plasma sintered Si_3_N_4_ ceramic matrix was enriched by multilayered graphene or graphene oxide to study the influence of the addition on mechanical, tribological, and electrical properties. The addition of multilayered graphene caused higher hardness, modulus, and bending strength in comparison to graphene oxide addition. However, the addition of graphene oxide and multilayered graphene resulted in lower mechanical properties but better electrical and tribological properties [172].

### 7.5. Building Materials and GO

Ordinary Portland cement (OPC) is one of the most used materials in the field of civil engineering thanks to the thirst for urbanization. The concrete is produced by mixing aggregates, binder (OPC), and water for hydration. Concrete has its advantages, such as unique compressive strength, as well as disadvantages, including poor crack formation resistance or low tensile strength [173]. Researchers have attempted many times to enhance the properties of cement-based materials by admixtures [174,175,176], fibers [177,178], and supplementary cementitious materials [179,180]. In more recent studies, newly produced nanomaterials such as nano-titanium oxide, nano-silica, nano-iron oxide, carbon nanotubes, and graphene oxide have been incorporated into the cement-like structures to enhance the mechanical properties of such materials. Such nanoparticles are able to fill even the smallest pores in the cement, providing a compact structure. Since GO is a two-dimensional material, it offers a large surface area for C-S-H nucleation [181,182]. The large surface area and the presence of functional groups make GO a highly reactive material. Mechanical properties of graphene are degraded by functionalization, meaning that GO shows lower elastic modulus and tensile strength than graphene. However, GO’s tensile strength and elastic modulus are still superior to those of cement—adding GO to cement-like materials enhances the mechanical properties of such building materials. Introducing small amounts of GO (0.05 wt.%) increases the flexural strength by 40–60% and compressive strength by 15–33% [182].

**Figure 9:**
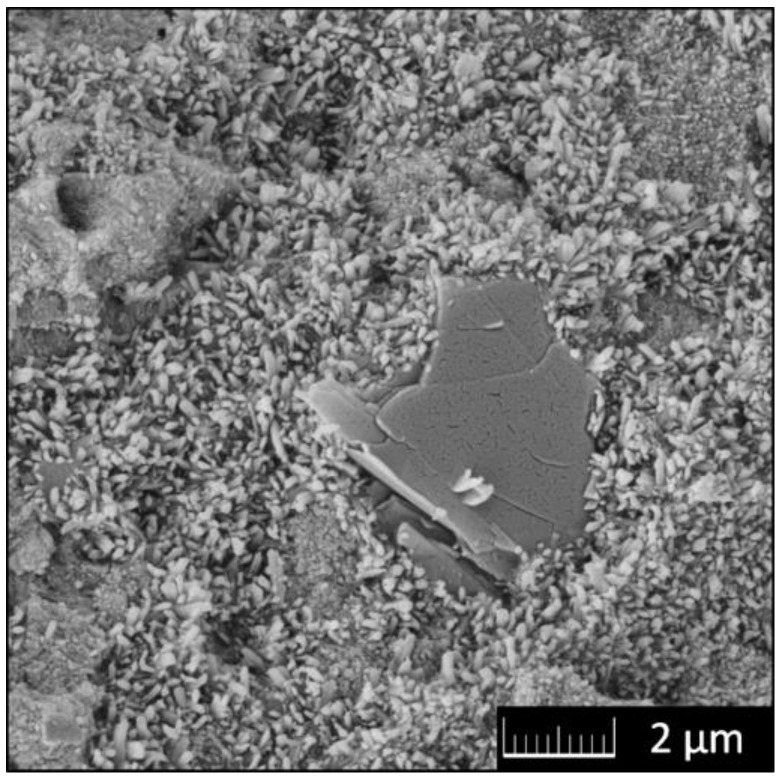
SEM micrograph of MOC-GO composite.

Magnesium oxychloride cements (MOCs) are promising alternatives to Portland cement. Let us note that OPC production is connected to high emissions of CO_2_ during manufacturing [183]. An alternative building material that can reduce the impact of CO_2_ during the carbonation is magnesium oxychloride cement (also known as Sorel cement) [184]. In order to enhance its flexural and compressive strength, carbonaceous nanomaterials, such as graphene, graphene oxide, or graphite oxide, can be added to the mixture (see Figure 9) [97]. Even the very poor water resistance can be improved by the addition of graphene [185].

## 8. Conclusions

In this article, the history, synthesis, properties, and application of graphene oxide were reviewed. There are many GO derivatives, including graphene acid, fluorographene, and graphene oxide reduced by thermal or chemical reduction (TRG or CRG). Graphene oxide as well as its derivatives have plenty of various applications. Thanks to the oxygen functional groups on the edges and basal plane, GO can be used as a solution for environmental problems such as excess of CO_2_ and toxic gases such as ammonia, acetone, formaldehyde, H_2_S, SO_2,_ and NO_x_. There is also a possibility to convert CO_2_ by photocatalytic reaction using GO-based composites. Organic dyes and inorganic heavy metal ions in water represent another worldwide environmental issue that can be solved by using GO. There are also medical applications such as gene delivery, which is a very promising way of treating genetic disorders; drug delivery for targeting cancer; and GO-based biosensors. GO-based membranes can be employed as molecular and ionic sieves or for selective gas transport. In high-temperature materials, GO works mainly as an additive used for reinforcement and general enhancement of mechanical properties. Employing GO into building materials such as ordinary Portland cement or magnesium oxychloride can enhance their flexural and compressive strength as well as MOC’s poor water resistance.

## Data Availability

The data presented in this study are available on request from the corresponding author. The data are not publicly available due to privacy.
